# Dissimilar autogenous TIG joint of Alloy 617 and AISI 304H steel for AUSC application

**DOI:** 10.1016/j.heliyon.2023.e19945

**Published:** 2023-09-07

**Authors:** Sachin Sirohi, Amit Kumar, Shailesh M. Pandey, Priyambada Purohit, Dariusz Fydrych, Sanjeev Kumar, Chandan Pandey

**Affiliations:** a Department of Mechanical Engineering, SRM Institute of Science and Technology, Delhi NCR Campus, Modinagar, 201204, Uttar Pradesh, India; b Department of Mechanical Engineering, Indian Institute of Technology Jodhpur, Jodhpur, 342037, Rajasthan, India; c Department of Mechanical Engineering, National Institute of Technology Patna, Patna, 500078, India; d Faculty of Management Studies, SRM Institute of Science and Technology, Delhi NCR Campus, Modinagar, 201204, Uttar Pradesh, India; e Institute of Manufacturing and Materials Technology, Faculty of Mechanical Engineering and Ship Technology, Gdańsk University of Technology, Gabriela Narutowicza Street 11/12, 80-233 Gdańsk, Poland

**Keywords:** Autogenous TIG, Dissimilar, Alloy 617, Impact toughness, Microstructure

## Abstract

To reduce costs and improve high-temperature performance in Advanced Ultra Super Critical (AUSC) boilers, it is necessary to weld austenitic steel to Inconel alloy. In this study, the autogenous tungsten inert gas (TIG) welding process was used to join Alloy 617 and an austenitic AISI 304H steel plate of thickness 5 mm. Microstructural analysis showed that the microstructure formation was uneven along the weldments, with columnar and cellular dendrites near the interface while the central area of the weld exhibited a combination of columnar, cellular, and equiaxed dendrites. The use of energy dispersive spectroscopy and electron probe micro-analysis unveiled the presence of an unmixed layer at the interface between the weld and AISI 304H steel. Furthermore, a notable variation in the concentration of alloying elements such as Fe, Cr, Ni, Co, and Mo was observed. Within the weld metal, inter-dendritic areas showed the presence of precipitates rich in Cr, Ti, and Mo. Meanwhile, the heat-affected zone (HAZ) of Alloy 617 exhibited the presence of phases like Cr and Mo-rich M_23_C_6_ as well as Mo-rich M_6_C. Hardness tests showed non-uniform hardness along the weldments, with a hardness of 199 ± 6 HV in the weld metal and 225 ± 4 HV in Alloy 617 HAZ, and 207 ± 7 HV in AISI 304H HAZ. The Mo and Cr segregation in the inter-dendritic spaces led to a decline in the tensile properties of the welded parts and resulted in failure from the region of the weld metal.

## 1 Introduction

Power production plants around the world primarily use gas, oil, and coal as fuels. Coal is a popular fuel source because it is inexpensive, but it is also a major source of CO_2_ emissions, which can lead to environmental issues like global warming [1]. To address this concern, power plants must reduce CO_2_ emissions by improving their efficiency. Advanced Ultra-Supercritical (AUSC) power plants have been developed to achieve higher efficiency (over 48%) with lower CO_2_ emissions [2]. To increase efficiency, the steam inlet temperature must be raised. The AUSC power plants can operate at temperatures up to 700 °C and under 350 bar pressure. Superalloys and heat-resistant steels have been developed to withstand the high temperatures of the AUSC power plants, including ferritic/martensitic grade, austenitic grade steels, and Ni-based superalloys. The Ni-based superalloys can withstand temperatures up to 700–760 °C, while advanced austenitic steel can work at temperatures around 620–680 °C [3]. The utilization of dissimilar metal welding (DMW) between the Ni-based Inconel 617 alloy and the austenitic grade AISI 304H steel is essential for the advancement of efficient and economically viable AUSC power plants, aiming to achieve heightened thermal efficiency [4]. Due to its elevated chromium content, AISI 304H steel shows exceptional corrosion and oxidation resistance, along with commendable creep strength. Additionally, its high chromium content contributes to its ease of processing and welding, enhancing overall performance in these aspects [5].

Inconel 617 alloy is a Ni-based austenitic superalloy that has exceptional creep strength. It is a solid solution-strengthened alloy which exhibits exceptional resistance to oxidation at elevated temperatures, coupled with favorable mechanical properties. Consequently, it emerges as an appealing choice for incorporating into high-temperature operating components in various industries, including power plants, nuclear, aerospace, and chemical industries [6]. The alloy offers impressive resistance to both oxidation and corrosion, maintaining its effectiveness up to temperatures as high as 1093 °C. Moreover, it shows notable creep resistance within the temperature range spanning from 650 °C to 1093 °C. The alloy's composition includes elements such as Mo, Co, Cr, and Al, which contribute to its strength and corrosion resistance [7]. However, the alloy is expensive due to its alloying composition, and an inexpensive material such as austenitic grade stainless steel, which has good mechanical properties and can resist temperatures of up to 650–680 °C, can be used as a substitute for lower-risk sections that operate at lower temperatures. The austenitic grade stainless steel (such as AISI 304 L, AISI 304H, and AISI 310) is a viable replacement for Inconel 617 alloy. To improve the structural integrity and reduce overall cost, a combination of Inconel 617 and cheaper SS-grade steel is being used in AUSC boilers. The joining of dissimilar alloy for AUSC application is mainly performed using the radiant beam welding processes and arc welding processes. Recently few works has been published on radiant beam welded joint for AUSC application [[8], [9], [10], [11]]. The welded joint produced using the radiant beam welding process meet the standard requirement of high temperature application but processes are costlier than the arc welding processes. Gas tungsten arc welding (GTAW) is used to join these dissimilar materials, but this process presents challenges due to the different chemical composition and thermal expansion coefficients of the base materials [12,13]. These challenges result in residual stresses, unmixed zones, and element diffusion across the fusion line [14,15]. The utilization of Ni-based filler metal offers a potential solution to these challenges. However, the segregation of alloying components may result in diminished impact toughness, a decline in ductility, susceptibility to crack formation, and reduced resistance to hot cracking [16,17]. Hosseini et al. [18] conducted a study on a GTAW joint between Inconel 617 and 310 SS plates using various fillers (IN617, IN82, and 310 SS). The IN617 filler demonstrated a favorable combination of tensile strength and impact toughness. In contrast, the 310 stainless steel filler displayed inadequate impact toughness and the presence of multiple cracks within the unmixed zone. In their study, Pavan et al. [4] examined the microstructure and stress rupture characteristics of a dissimilar weld joint comprising IN617 and SUS 304 H steel. They employed the GTAW process with an IN617 filler material for this investigation. Their findings revealed that the weld joint created using the IN617 filler material exhibited a favorable combination of tensile strength and impact toughness. This quality renders it well-suited for application in AUSC boilers. On the other hand, a study by Naffakh et al. [19] compared the use of IN617 and 310 SS fillers for the GTAW joint of 310 SS and Inconel 657. The IN617 filler resulted in dendritic microstructure, which made it more susceptible to embrittlement. In contrast, the 310 SS filler was more prone to hot cracking due to the formation of low melting phases. Conversely, the 310 SS filler was susceptible to hot cracking, primarily attributed to the creation of low melting phases. Opting for Inconel A filler emerged as the optimal choice to achieve dissimilar weld joints between IN657 and 310 SS. This preference was attributed to the narrow solidification range of Inconel A filler and its reduced tendency for the segregation of alloying elements. In a separate study conducted by Naffakh et al. [20], an examination of the weldability and susceptibility to hot cracking in IN657 and 310 SS dissimilar weldments was carried out using Varestraint tests. The outcomes revealed that Inconel A filler displayed the lowest susceptibility to hot cracking, in contrast to the substantial susceptibility observed with the 310 SS filler. According to research conducted by Mithilesh et al. [21], the utilization of a nickel-based filler (specifically IN625) proved to be a viable and appropriate option for producing a structurally sound joint between Inconel 625 and SS304L steel. In a separate examination involving a dissimilar joint composed of IN617 and P92 steel, the IN617 filler material demonstrated the highest strength and the least residual stresses, positioning it as the most favorable filler choice [2]. Similarly, for GTAW joints between IN718 and 310 stainless steel (SS), the IN82 filler emerged as the recommended option. This choice was based on its ability to yield optimal impact toughness and the highest potential for corrosion resistance. However, eliminating problems related to multi-pass welding, unmixed zone formation, element diffusion, inferior mechanical properties due to element segregation, and residual stresses remains challenging. In recent times, there has been an increase in the use of autogenous welding processes, i.e., radiant beam and autogenous and activated TIG for similar and dissimilar metal joining. These welding methods have helped to rectify some problems related to elemental diffusion across interfaces, inhomogeneous microstructure and mechanical properties in weld zone [14,[22], [23], [24]]. Vidyarthy et al. [22] performed the welding of 409 Ferritic Stainless Steel using a multi-pass activated TIG welding process and the results confirmed the less distortion and superior mechanical properties for the A-TIG welded joint. Sharma and Dwivedi [25] also conducted research on dissimilar joints of P92 and AISI 304 L steel fabricated using the A-TIG process. Full penetration is achieved for an 8 mm thick plate in a single A-TIG pass. The comparative analysis of the multi-pass TIG and A-TIG joint showed superior mechanical strength and cost-effective joint for A-TIG. In a study by Ramkumar et al. [10], an electron beam welded dissimilar joint consisting of Inconel 625 and UNS 32205 was analyzed for its microstructure and mechanical attributes. Tensile testing revealed that failure originated within the weld metal, attributed to the segregation of alloying elements. Moreover, the impact properties exhibited by the joint were notably lower than those of the base metals, primarily due to the segregation of alloying particles. Pandey [26] fabricated the dissimilar joint of the P92 and AISI 304 L plate of thickness 5.5. mm in a single run using an autogenous TIG process. Acceptable tensile and impact properties were confirmed from the mechanical testing. In the research conducted by Li et al. [27], an investigation was conducted on a welded joint of IN625 and SUS 304 produced using a high-power CO_2_ laser. The tensile test revealed that fracture initiation originated within the weld metal of the tested specimen. Furthermore, the impact test outcomes indicated a lower toughness value compared to the base metals (BMs). This decrease in toughness was attributed to the segregation of Nb and Mo within the weld metal.

The comprehensive review of the literature shows that dissimilar welding through the GTAW process presents a range of challenges. Notably, one of these challenges pertains to the critical decision-making process surrounding the choice of appropriate filler metal. However, autogenous welding processes do not face these issues and can be used successfully for making dissimilar joints with a plate thickness of up to 5 mm. Furthermore, there is limited research available on the dissimilar joining, characterization, and mechanical testing of Inconel alloy and austenitic grade AISI 304H steel. Additionally, there is no published research available on the autogenous TIG welding of dissimilar Inconel 617/AISI 304H for AUSC boiler applications. Consequently, the focus of this study is to bridge this research gap by delving into the intricate relationship between microstructure and mechanical properties of autogenous tungsten inert gas (TIG) dissimilar weldments of Inconel 617 and AISI 304H materials.

## 2 Materials and methods

The study utilized commercial-grade IN617 alloy (0.06% C, 0.4% Si, 0.45% Mn, 21.52% Cr, 12.4% Co, 8.9% Mo, 1.1% Al, 0.2% Ti, 1.3% Fe, and balance Ni) and austenitic grade AISI 304H steel (0.11% C, 0.42% Mn, 7.86% Ni, 0.24% Si, 19.3% Cr, 0.90% Mo, 0.42% Co, and balance Fe) plates, both machined to dimensions of 110 mm × 55 mm × 5 mm, to produce dissimilar weldments using an autogenous tungsten inert gas (TIG) welding power source. The mechanical properties of the base plates were evaluated using standard specimens of base metals and given in Table 1 [2,28]**.**

**Table 1:** Mechanical properties.

Mechanical properties	IN617 alloy	AISI 304H steel
Tensile strength (MPa)	775 ± 7	640 ± 4
Yield strength (MPa)	235 ± 3	240 ± 3
Microhardness (HV)	217 ± 2	198 ± 6
Impact toughness (J)	142 ± 3	285 ± 5
Elongation (%)	82 ± 3	96 ± 4
Reduction in the area (%)	76 ± 3	50 ± 4

**Fig. 1:**
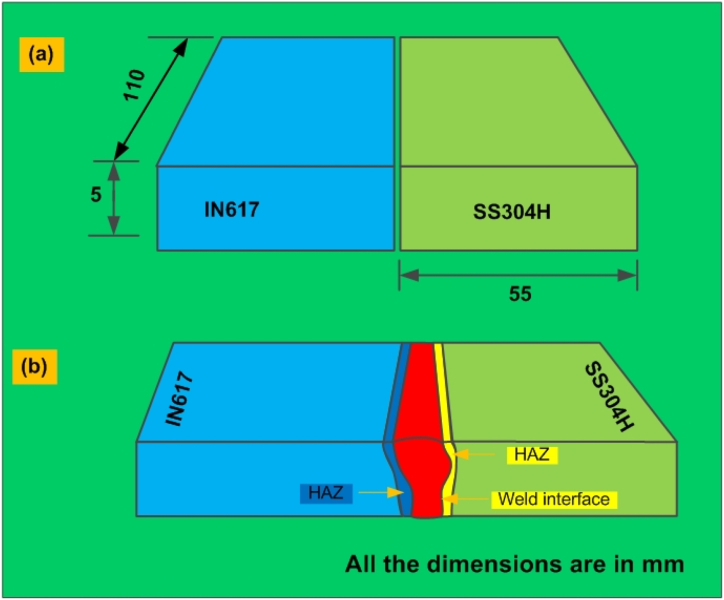
(a) Schematic of the base plates were arranged for joint preparation, (b) weldments of IN617 and AISI 304H plate.

**Fig. 2:**
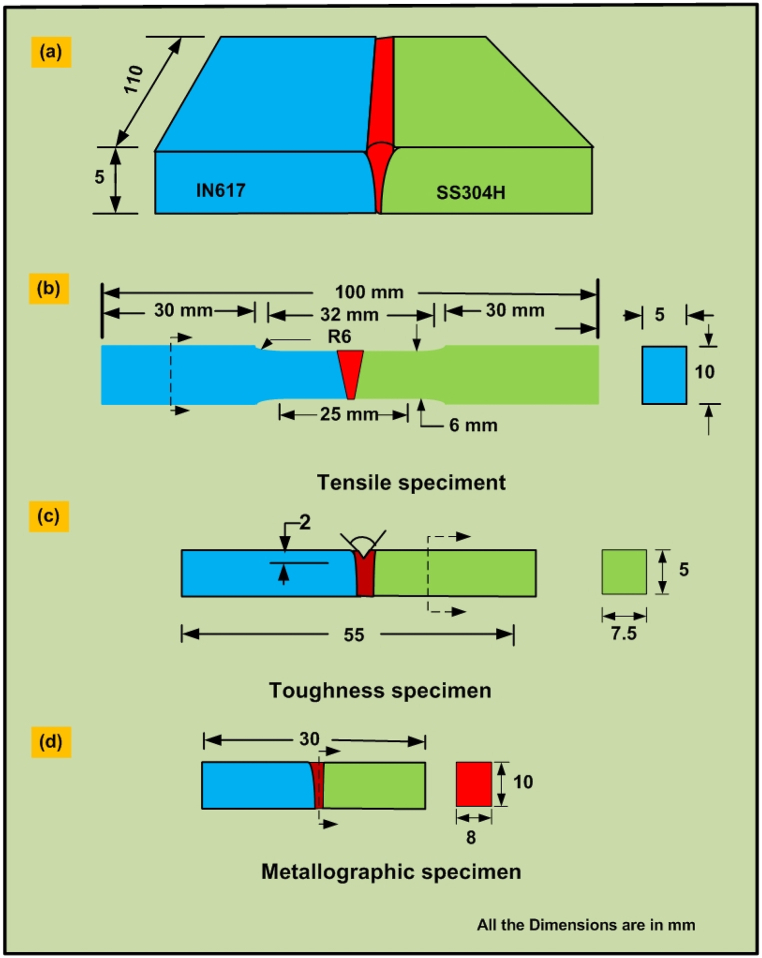
Schematic of mechanical test specimens machined according to the guidelines outlined in the ASTM standard.

The edges of the plates were first machined using a shaper and then further smoothed through grinding using a grinding machine. Following this, SiC emery paper was employed to polish the plates. In preparation for welding, the plates underwent cleaning using acetone, which effectively removed surface rust and dust particles. The schematic depiction of the base plates used for joint preparation can be seen in in Fig. 1(a). To prepare the joint, welding was conducted at a current of 200 A, with an arc voltage of 12 V and a travel speed of 80 mm/min. The electrode angle was set at 60°, and the arc length was maintained at 3 mm. To avert the entrapment of atmospheric gases, pure argon with a high purity level of 99.99% was employed. A glimpse into the microstructural features along the weldments is presented in in Fig. 1(b). For mechanical testing purposes, specimens were obtained through wire-cut electro-discharge machining (EDM). The extraction design employed for assessing the structural integrity is depicted in Fig. 2(a). After the welding process was finalized, samples were machined for the purpose of both mechanical testing and microstructural analysis. A specimen measuring 30 mm × 10 mm x 8 mm (Fig. 2(d)) was cut from the sample using wire-cut EDM and subjected to mechanical polishing with various grit sizes of emery paper before being polished with alumina powder using a disc polishing method. The microstructure examination of the weld metal and HAZ in IN617 involved a multi-step process. The polished samples underwent ultrasonic cleaning, followed by electrochemical etching using oxalic acid to reveal the microstructure. Similarly, on the opposite side HAZ (AISI 304H), etching was carried out using an aqua regia solution. To analyze these microstructures, optical microscopy (OM) and scanning electron microscopy (SEM) were employed. Optical microscopy was conducted using a Leica DMC4500 model, while SEM examinations utilized the Carl Zeiss Ultra plus and FEI Quanta 200 instruments. These examinations covered various regions within the weldments, facilitating the investigation of element migration across interfaces and enabling the analysis of phase composition within the weld metal and HAZ. This analysis was facilitated through electron probe microanalysis (EPMA) and electron dispersive X-ray spectroscopy using SEM. Tensile, impact toughness and hardness test specimens were then extracted in accordance with ASTM standards to establish the microstructure-property relationship. Tensile tests were performed on a vertical tensile testing machine (Instron 5980 of 100 kN capacity, Instron, MA, USA) at a constant strain rate of 6.6 × 10^−4^/sec as per ASTM E8M (Fig. 2(b)) [29]. To assess the hardness distribution across the transverse coupons of the welded joint, a Vickers Micro-hardness tester (Autovick HM-200) was employed. The hardness analysis involved applying a load of 500 g with a 10 s dwell time. For evaluating the impact strength of the weldments, V-notch Charpy impact specimens were fabricated in accordance with the ASTM E23-02a standard [30] (as depicted in Fig. 2(c)). A Charpy impact test was conducted at room temperature on the weld metal. This evaluation was carried out using a Charpy impact tester (FIT-400-ASTM-D, Fine Testing Machines Pvt. Ltd., Miraj, India). In order to ensure the consistency and reliability of the results, three samples were subjected to impact tests for each trial.

## 3 Results and discussion

### 3.1 Base material

**Fig. 3:**
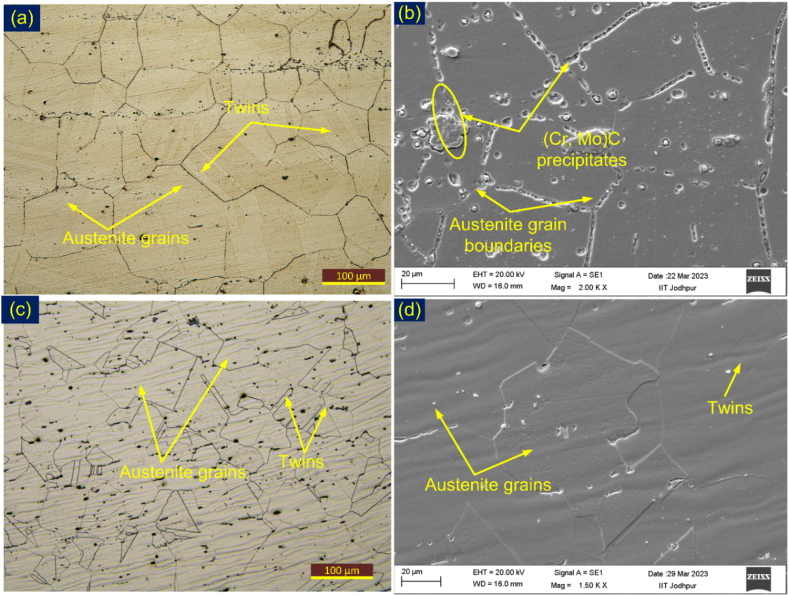
Microstructure (a, b) IN617 alloy, (c, d) AISI 304H steel.

The IN617 alloy exhibited a fully austenitic microstructure containing varying sizes of austenite grains and annealing twins, as depicted in Fig. 3(a). The SEM images (Fig. 3(b)) revealed a heterogeneous distribution of precipitates of varying size and shape, with Cr-rich M_23_C_6_ and Mo-rich Mo_6_C being located along the boundaries and within grains, and Ti(C, N) being situated at the intra-granular position [31]**.** A similar observation has been made in previous work also [32]. The EDS results presented in Table 2 confirmed the presence of M_23_C_6_, Mo_6_C, and Ti(C, N) precipitates in the IN617 base material. The austenitic matrix of AISI 304H exhibited an austenitic microstructure with equiaxed polygonal grains and twins, as shown in Fig. 3(c) and (d). The average austenite grain size was found to be 72 ± 15 μm for IN617 and 30 ± 3 μm for AISI 304H BM.

**Table 2:** EDS results (wt.%).

Phases in Inconel 617	C	Si	Ti	Cr	Mn	Fe	Co	Ni	Mo
M_23_C_6_	28.45	0.79	0.24	41.58	0.32	0.16	1.85	13.09	13.52
TiC	8.52	0.12	61.87	5.82	–	0.68	3.85	10.52	8.62
Mo_6_C	38	0.45	0.36	12.45	0.28	0.42	6.62	13	28.42
Matrix Inconel 617 BM	–	0.15	0.24	20.38	0.21	1.94	11.25	55.75	10.23
	C	Si	S	Cr	Mn	Fe	Ni	–	–
Matrix 304H SS	3.2	0.28	0.06	17.32	0.84	71.48	6.82	0	0

### 3.2 Characterization of dissimilar weldments

#### 3.2.1 Interface and HAZ

**Fig. 4:**
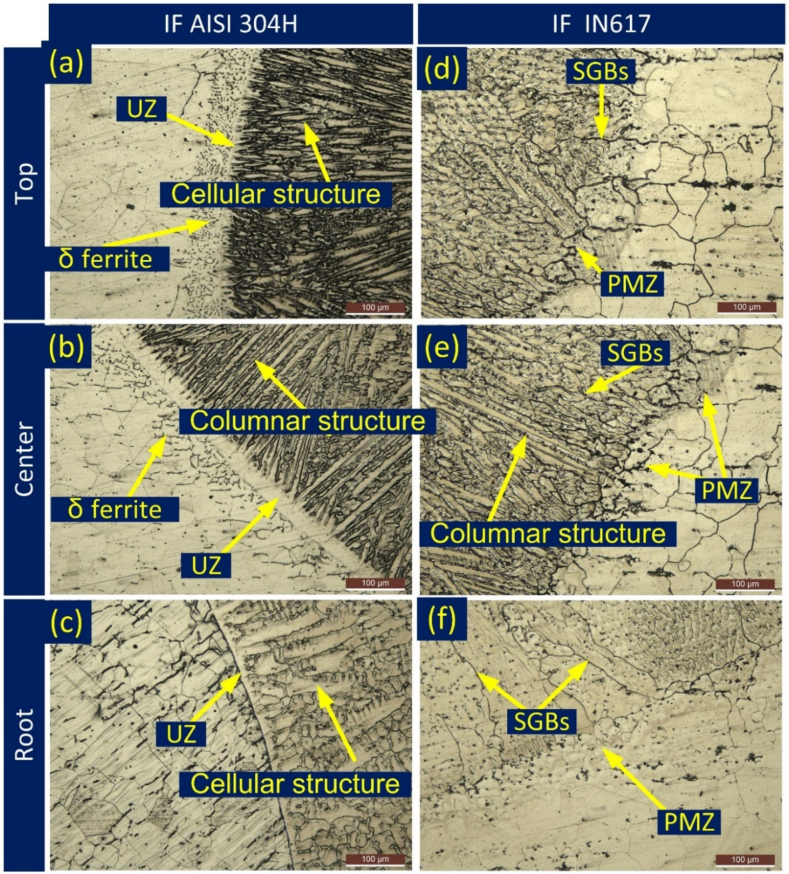
Interface image from different locations (a–c), on AISI 304H side (d–f) IN617 side (UZ: Unmixed zone; PMZ: Partially melted zone; SGB: solidified grain boundary).

The macrograph indicates that there are no observable cracks present during or after welding, and the interfaces are strongly bonded without any defects. Microstructures at various locations on both sides of the narrow weld bead were captured at the interface region. Based on the interfacial microstructure, it can be concluded that there is no noteworthy alteration in the microstructure near the fusion line. The depiction of the interface reveals the presence of cellular and columnar growth patterns in the weld metal vicinity near the fusion line. The weld metal adjacent to the IN617 BM displays a more refined structure when contrasted with the weld metal neighbouring the AISI 304H BM. In the vicinity of the IN617 interface, a partially melted zone (PMZ) is noticeable, accompanied by a minimal unmixed zone (UZ), as depicted in Fig. 4(d and e). Conversely, a significant width of UZ (Fig. 4(a, b)) is observed parallel to the fusion line on the AISI 304H side. A very narrow section of UZ was visible in the root area (as shown in Fig. 4(c)), as well as a narrow section of PMZ (as shown in Fig. 4(f)). Within the interface area of AISI 304H, the presence of both a PMZ and a UZ is evident. The occurrence of the PMZ is attributed to the segregation of low melting impurity elements in proximity to the grain boundaries. Additionally, noticeable thickening of the grain boundaries was observed within the PMZ, as depicted in Fig. 4(d–f). The region of AISI 304H BM was adjacent to the fusion line and did not exhibit any significant grain growth (Fig. 4(a-c)).

**Fig. 5:**
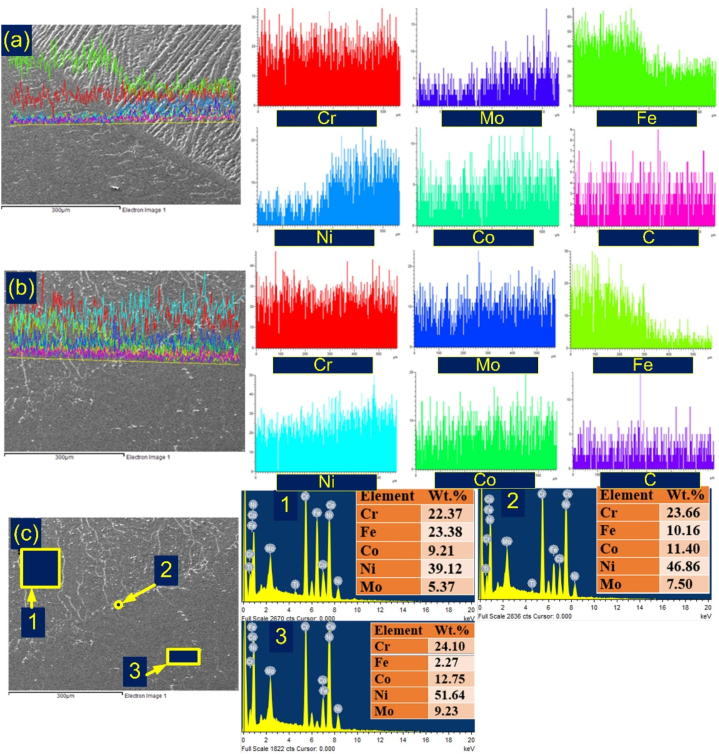
Line map of the weld interface of (a) AISI 304H, (b) IN617, (c) IN617 side with EDS.

Conversely, a notable extent of grain growth was observed at the interface of IN617/weld metal, accompanied by the emergence of an exceedingly narrow or practically non-existent PMZ, as illustrated in Fig. 4(d–f). The phenomenon of epitaxial grain formation within the weld metal, coupled with the absence of an UZ near the interface, can be attributed to the similarity in elemental composition and microstructure between the IN617 BM and the weld metal. However, it's worth noting that the segregation of alloying elements was detected at the interface and within the confined PMZ (Fig. 4(d-f)). In-depth examination of the interface region was conducted using SEM/EDS analysis, with the outcomes presented in Fig. 5.

Fig. 5 presents the observation of the EDS line map. The migration of Fe from AISI 304H to the weld metal is depicted in the EDS line map presented in Fig. 5(a and b). This map also illustrates the migration of Ni, Co, and Mo from IN617 BM to the weld metal. However, the most significant difference was observed for Ni and Fe at the interface of the weld metal and AISI 304H. The line map study covered the IN617/weld metal interface and demonstrated a significant increase in Fe intensity in the vicinity of the IN617 HAZ. In Fig. 5(c), the interface area present on the IN617 side is illustrated. The marked points, as shown in Fig. 5(c), were used to perform EDS analysis. Comparatively, the weld metal exhibited reduced weight percentages of Ni, Cr, and Co in contrast to the analogous IN617 BM. This could be attributed to the potential mixing of these two materials (EDS area 1 and EDS area 3). The EDS of the weld (EDS area 1) shows the major concentration of Cr, Fe, Co, Ni and Mo. At the interface point, the concentration of the Fe (10.16%) was measured lower than weld (23.38%) but higher than IN617 BM (2.27%). The Cr, Co and Mo concentrations also measured higher at the interface than at the weld.

**Fig. 6:**
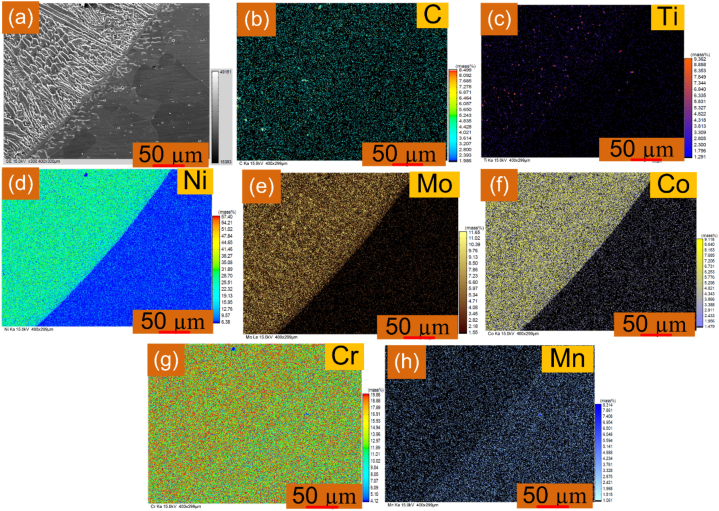
EPMA of interface form along AISI 304H interface (a) grey scale image; element distribution: (b) C, (c) Ti, (d) Ni, (e) Mo, (f) Co, (g) Cr, (h) Mn.

**Fig. 7:**
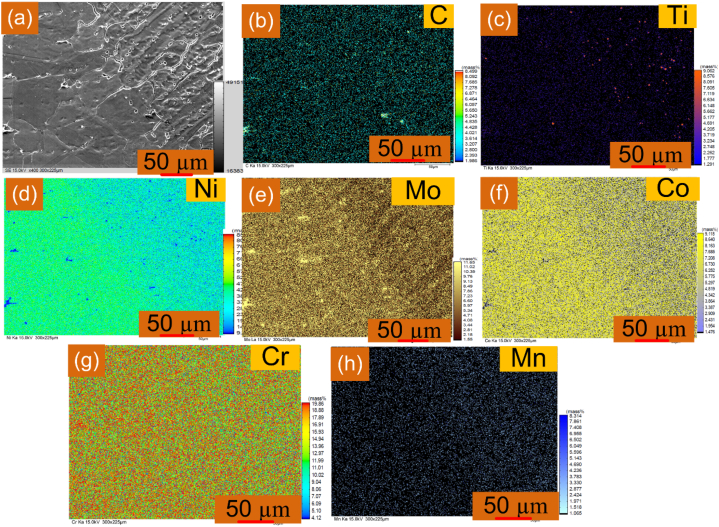
EPMA of interface form along IN617 interface: (a) grey scale image; element distribution: (b) C, (c) Ti, (d) Ni, (e) Mo, (f) Co, (g) Cr, (h) Mn.

Additionally, the EPMA study has been carried out on both sides of the weld metal and is shown in Fig. 6, Fig. 7. For the EPMA study, a grey-scale SEM image was used (Fig. 6, Fig. 7). The interface of AISI 304H is presented in Fig. 6(a–h). The findings show that Ti is more concentrated (Fig. 6(c)) in the solidified weld metal, and there is a chance that the phase is Ti(C, N). The presence of the Mo-rich phases was supported by the fact that the concentration of Mo also varies in the weld metal close to the interface (Fig. 6(e)). This might be Mo_6_C or Cr and Mo-rich M_23_C_6_. Weld metal has a higher content of Co, Ni, Cr, and Mo than AISI 304H BM (Fig. 6(d-g)). A slight rise in the concentration of carbon at the interface was found, and it might be the result of carbon diffusing from AISI 304H to weld metal. The Mn and Cr are particularly concentrated in the 304H SS. The interface of IN617 is presented in Fig. 7(a–h). It confirms the major phase of the Cr and Mo in IN 617 BM (Fig. 7(e, g)). The interface region confirms the diffusion of Co, Cr, Mo, and Ni (Fig. 7(d-g)). The IN617 BM has a higher concentration of Ti (Fig. 7(c)) at some points and it might be the phase of the Ti(C, N). However, the weld metal near the interface shows a higher density of the Ti(C, N).

**Fig. 8:**
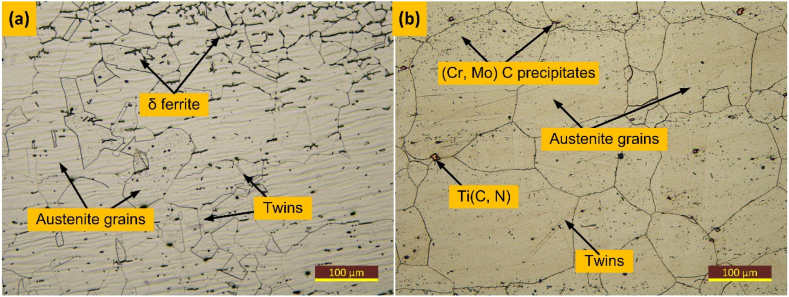
HAZ of AISI 304H and IN617 are shown in (a) and (b), respectively.

Fig. 8 displays the HAZ along the fusion line for both IN617 and AISI 304H BMs. Although there was grain growth seen along the fusion line due to the intense thermal cycle, the grain size in the AISI 304H HAZ was found to be similar to the BM. The size of the austenite grains ranged from 25 μm to 55 μm, with an average measurement of 32 ± 7 μm. Fig. 8(a) also presents instances of twins, lath ferrite stringers, and polygonal equiaxed austenite grains. One significant component that facilitates the formation of the ferrite stringer is the segregation of ferrite stabilizing elements like Cr during the solidification process [33]**.** In contrast, significant grain growth was observed in the IN617 HAZ (Fig. 8(b)), with a grain size ranging from 49 μm to 153 μm with an average of 99 ± 27 μm. The average austenite grain size was 72 ± 15 μm for IN617 base metal. In their study, Hossein et al. [18] also documented grain enlargement within the HAZ adjacent to the fusion line in the fusion-welded joint involving IN617 and 310SS materials.

**Fig. 9:**
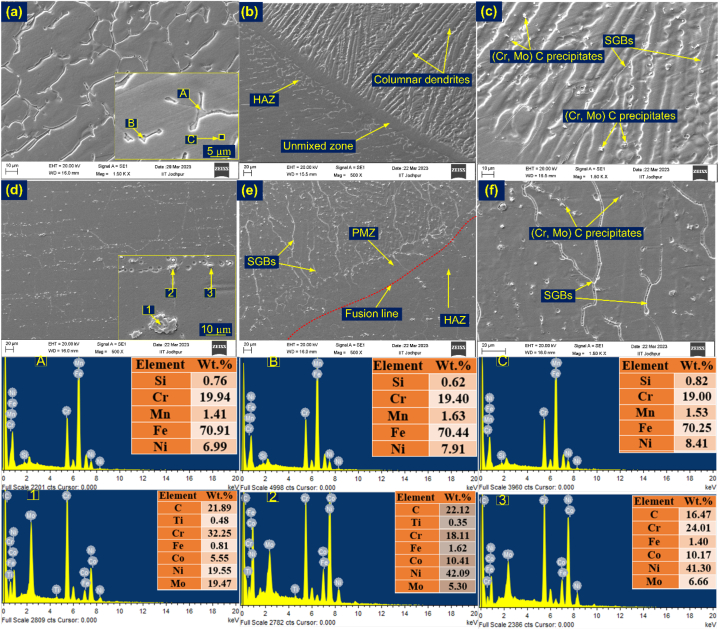
(a) SEM image of IN617 HAZ and marked EDS location (b) interface of AISI 304H side, (c) weld near interface of AISI 304H, (d) IN617 HAZ and marked EDS location, (e) interface of IN617 side, (f) weld near interface of IN617.

Fig. 9(a) shows the SEM image of HAZ of AISI 304H, revealing the presence of lathy δ ferrite. The distribution of major elements in the HAZ matrix (EDS point C), including Cr (19.0 wt%), Mn (1.53 wt%), Fe (70.25 wt%), and Ni (8.41 wt%), was determined using EDS analysis, and the corresponding spectra are presented in Fig. 9. The EDS of the ferrite (EDS point A and B) shows the major concentration of the Cr (19.40–19.94 wt%), Mn (1.41–1.63 wt%), Fe (70.44–70.91 wt%) and Ni (6.99–7.91 wt%). The image displayed in Fig. 9(b) depicts the interface between AISI 304H and weld metal. It verifies that the weld metal near the interface has columnar grain growth, which is also evident in the optical image showing the formation of the UZ. Additionally, the ferrite density was found to be greater in the HAZ of AISI 304H adjacent to the interface. Fig. 9(c), provides a closer look at the weld metal in the vicinity of the interface. This image displays the solidified grain boundaries (SGBs) and the separation of alloying elements within the inter-dendritic spaces. Fig. 9(d) presents an SEM image of the HAZ in IN617, which revealed the presence of both inter and intra-granular precipitates. Both coarse and fine carbide phases were evident both along the austenitic boundaries and within the matrix. Notably, in the HAZ, there was a progression of lamellar carbides forming at the grain boundaries. This phenomenon led to comparatively weaker connections between the grains, differing from the behavior observed in the IN617 BM. Furthermore, the size of the carbide phases was observed to be larger than those present in the IN617 BM. This could be attributed to the influence of the elevated temperatures generated by the multi-pass welding process. The optical image also confirmed the presence of both coarse and fine carbide phases in the HAZ. EDS analysis was conducted on the white particles to determine their elemental composition, as shown in Fig. 9. The results indicate the presence of the Cr and Mo-rich M_23_C_6_ secondary phase in the bulky particles (EDS spot 2), which are abundant in Cr (18.11 wt%) and Mo (5.30 wt%). The coarse spherical particles (EDS spot 1) exhibit a primary concentration of Mo, which is a 19.47 wt percentage and could potentially be the Mo-rich M_6_C phase. The EDS analysis of the fine spherical particles (EDS spot 3) indicates a high concentration of Cr and Mo, suggesting the presence of Cr and Mo-rich M_23_C_6_ secondary phase precipitates. The interface on IN617 side and weld metal near the interface is depicted in Fig. 9(e and f).

#### 3.2.2 Weld metal

**Fig. 10:**
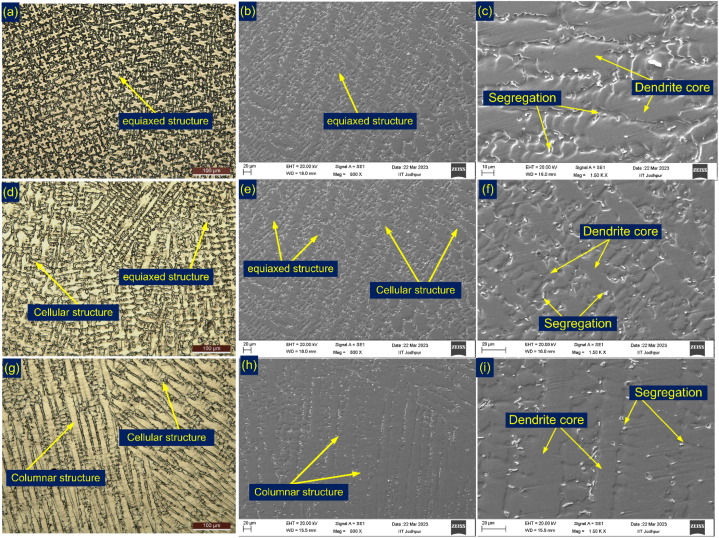
Weld metal from the top (a–c), centre (d–f), and root (g–i) portions to reveal the variation of microstructure.

The weld metal exhibits a diverse microstructure, which is evident from Fig. 10. The alteration in cooling rates across the top to root weld zones led to variations in solidification modes, resulting in the formation of a heterogeneous microstructure within the weld metal. The disparities in thermo-physical properties of the two BMs are primarily accountable for the differences observed in the microstructure of the weld metal. The predominant solidified microstructure within the weld metal is determined largely by the extent of constitutional supercooling, represented as the ratio of temperature gradient (G) to growth rate (R). Additionally, the cooling rate is approximated as the product of G and R. As illustrated in Fig. 10(a–c), the top region of the weld displays an equiaxed dendritic microstructure. This region is also characterized by the segregation of alloying particles, as shown in Fig. 10(b and c). The majority of the weld metal's center region is covered with equiaxed and cellular structures (Fig. 10(d–f)). This region further displays the segregation of elements in the inter-dendritic areas, as depicted in Fig. 10(d–f). In the narrow root region, the image captured in Fig. 10(g) showcases a substantial portion of columnar dendrites. The dendrite core and precipitates located along the boundaries are shown in Fig. 10(h and i).

**Fig. 11:**
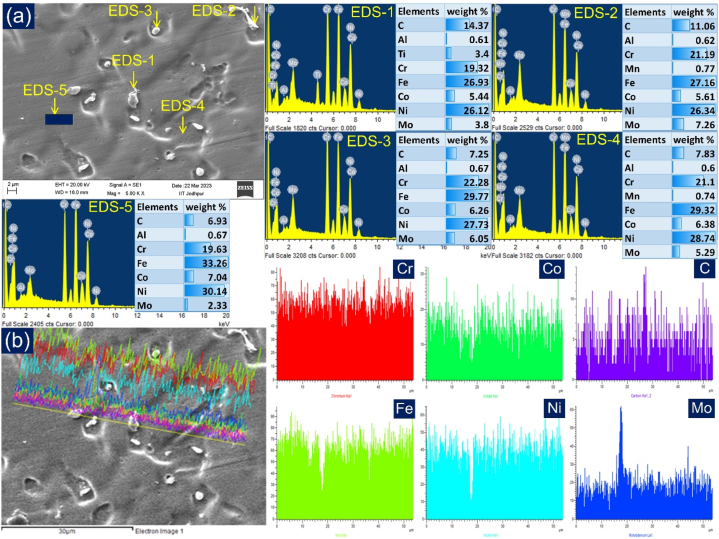
(a) Weld metal along with the marked location of EDS and their results, (b) elemental line map across the dendrite core and boundaries.

Fig. 11(a) illustrate the weld metal micrograph in the centre region. The equiaxed structure in the central region is due to uniform and symmetrical cooling, unlike the interface region. The image provides insight into the existence of secondary phases within the inter-dendritic regions of the austenitic matrix (Fig. 9(a)). The accompanying line map illustrates that Ni, Co, Fe, and Cr constitute the primary elements found at the dendrite core. In contrast, Mo and C are predominant elements located along the inter-dendritic spaces (Fig. 11(b)). The phases occupying the inter-dendritic spaces exhibit a notable higher concentration of Mo (wt.%) as evidenced by the results of point EDS analysis. Fig. 11(a) shows an SEM micrograph of the weld metal with marked EDS locations. The EDS analysis highlights that as a consequence of Mo and Cr segregation during solidification, the phases within the inter-dendritic regions exhibited higher concentrations of Mo and Cr compared to the surrounding matrix. In contrast, the dendrite core displayed enrichment in Ni, Co, and Fe. Furthermore, spot EDS analysis indicates that the composition of the weld metal is a composite of both IN617 and AISI 304H BMs.

The precipitates located in the inter-dendritic spaces are likely Mo and Cr-rich M_23_C_6_ and M_6_C carbide phases (EDS 1, 2 and 3). The EDS analysis reveals that the inter-dendritic region (EDS 4) has a higher concentration of Mo, Cr, and C compared to the dendrite core (EDS 5). Research conducted on electron and laser beam dissimilar joints has proposed that the secondary phase particles present in the weld metal originate from Ni-based alloys, such as IN617 and IN625 [10,[34], [35], [36]]. The primary factor behind the emergence of secondary phases in the weld metal is the segregation of particles. Rapid cooling subsequent to solidification within the austenitic matrix of the weld metal leads to the formation of these secondary phases. The observation is consistent with earlier observations by Ramkumar et al. [10] and Ren et al. [37]. Previous research has also documented the segregation of elements in the fusion welding of steel and Ni-based alloys [17,38,39]. Naffakh et al. [40] identified NbC particles in the inter-dendritic regions of the IN657/AISI 310 joint. The segregation of Nb, Mo, and Cr elements was also observed in GTAW joints of dissimilar metals, namely Incoloy 800 and P91 [41,42]. Moreover, the secondary phases identified in the weld metal exhibited comparable morphology and composition to those found in the IN617 BM.

**Fig. 12:**
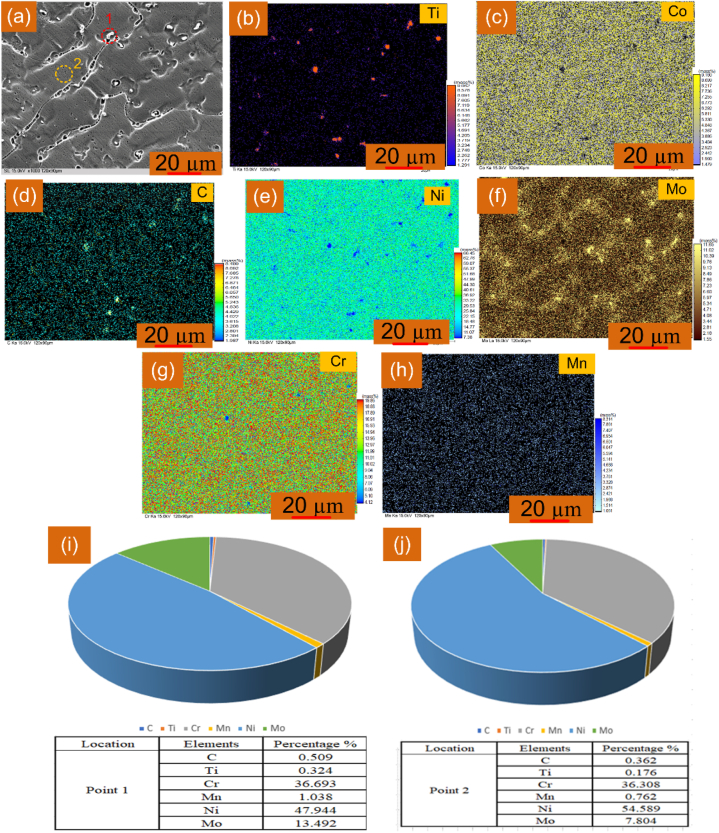
(a) Grey scale image selected for EPMA and EDS; EPMA map showing distribution of (b) Ti, (c) Co, (d) C, (e) Ni, (f) Mo, (g) Cr, (h) Mn, (i) EDS of white precipitates, (j) EDS of dendrite core.

The weld metal image selected for the EPMA study is displayed in Fig. 12(a). The EMPA results (Fig. 12(b-h)) confirm the major concentration of Mo in inter-dendritic areas and it would be the phase of Mo_6_C or M_23_C_6_. The same has also been confirmed in the EDS study presented in Fig. 11. The EDS in EPMA study also confirmed the higher concentration of the Mo at inter-dendritic space (Fig. 12(i)) than dendrite core (Fig. 12(j)).

### 3.3 Mechanical properties

#### 3.3.1 Micro-hardness

**Fig. 13:**
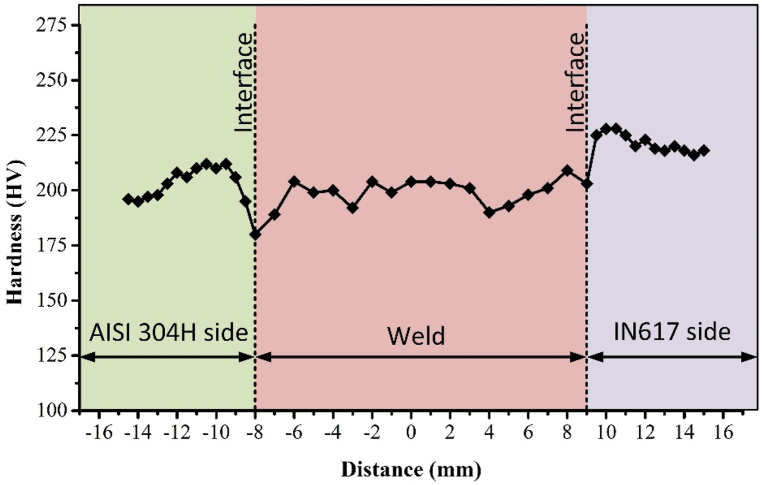
Hardness plot along weldments.

Fig. 13 displays the results of micro-indentation hardness measurements. The weld metal had an average hardness of 199 ± 6 HV in the transverse. This was found to be closer to the hardness of the AISI 304H base metals (198 ± 6 HV), but lower than the IN617 base metal (217 ± 2 HV). According to the metallographic section's discussion, the formation of dendritic structures and the segregation of alloying elements like Cr and Mo are responsible for the weld metal's increased hardness. The variation in hardness along the thickness was attributed to the microstructural inhomogeneity caused by changes in heating and cooling rates, resulting in the production of columnar, cellular, and equiaxed dendrites. The weld metal's highest and lowest hardness measurements, 189 HV and 209 HV, respectively, showed a 20 HV difference in hardness**.** This variation in hardness was primarily attributed to the segregation of alloying elements, resulting in higher hardness at grain boundaries compared to the grain interior. Notably, the hardness exhibited a progressive increase from the AISI 304H BM to the IN617 BM. The heterogeneity in microstructure across the weldments emerged as the key contributor to the observed hardness variation. The hardness at the interface and HAZ of the AISI 304H side were 180 HV and 207 ± 7 HV, respectively, which was greater than that of AISI 304H BM (198 ± 6 HV). The sequence of hardness on the IN617 side was IN617 HAZ > IN617 BM > IN617 IF. The hardness plot showcased an extended HAZ region on the IN617 side. The elevation in hardness within the IN617 HAZ was attributed to the presence of recrystallization and high-hardness elements**.** Importantly, the interface of the AISI 304H steel was recognized as the weakest region within the weldments, characterized by a comparatively lower hardness, as indicated in Table 3.

**Table 3:** Hardness test results.

AISI 304H			Weld	IN617		
BM	HAZ	IF		IF	HAZ	BM
198 ± 6 HV	207 ± 7 HV	180 HV	199 ± 6 HV	203 HV	225 ± 4 HV	217 ± 2 HV

#### 3.3.2 Tensile properties

**Fig. 14:**
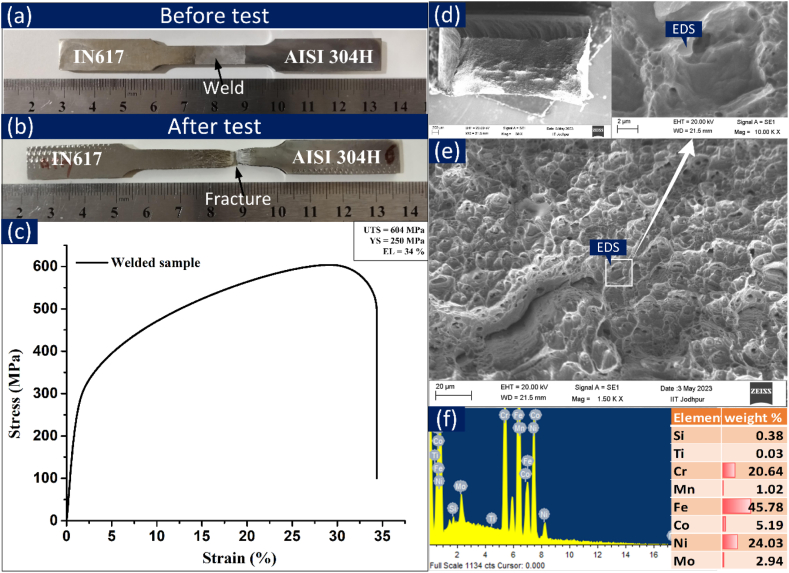
(a) Macrograph of tensile test specimens before fracture, (b) after fracture, (c) stress-strain plot for test specimens of the welded joint, (d) top view of the fracture surface, (e) detailed view, (f) EDS of marked point.

Fig. 14(a) displays the tensile test specimens marked with the location of the weld metal, while Fig. 14(b) shows the fractured specimen with the marked fracture location. The test specimens were fractured from the AISI 304H BM near to interface, and their average tensile strength was 604 ± 6 MPa. This value of tensile strengths of welded joint was measured lower than the AISI 304H BM (640 ± 4 MPa) and IN617 BM (775 ± 3 MPa). The order of tensile strength was as demonstrated: IN617 BM > AISI 304H BM > welded joint. Dissimilar welds fabricated using the TIG method are appropriate for elevated-temperature functionality in AUSC boilers. These tensile outcomes align with the findings of Pavan et al. [43], who investigated dissimilar welds between IN617 and SS304H produced using the GTAW technique and IN617 filler material. However, for a similar type of joint, obtained using the laser beam welding process, failure was reported in the region of the weld metal [32]. Fig. 14(c) displays the stress-strain graphs, juxtaposed with those of the BMs, to facilitate a comparison of the tensile properties between the weldments and the BMs. The weldments exhibited an elongation of 34%, which is significantly lower than the elongation of the BMs (IN617: 98% and AISI 304H: 84%). The acquired tensile characteristics for each test specimen are presented in Table 4, alongside the corresponding data for the BMs**.** The top view of the fractured surface is displayed in Fig. 14(d). The detailed view (Fig. 14(e)) shows the shallow dimples and microvoids along with cleavage area that confirms the mixed mode of the fracture. The secondary phase particles are also appeared at the fracture surface which are confirmed as Cr and Mo rich carbides from EDS analysis (Fig. 14(f)).

**Table 4:** Tensile characteristics of weldment.

Specimen	Tensile properties			Weld mismatch with AISI 304H	Weld mismatch with IN617 alloy	Failure region
	Tensile strength (MPa)	Yield strength (MPa)	% elongation			
IN617 alloy BM	775 ± 3	235 ± 3	96 ± 4	–	–	–
AISI 304H steel BM	640 ± 4	240 ± 3	82 ± 3	–	–	–
Welded specimen	604 ± 6	250 ± 4	34 ± 5	1.08	0.88	Weld metal near the interface of AISI 304H BM

#### 3.3.3 Impact toughness

**Fig. 15:**
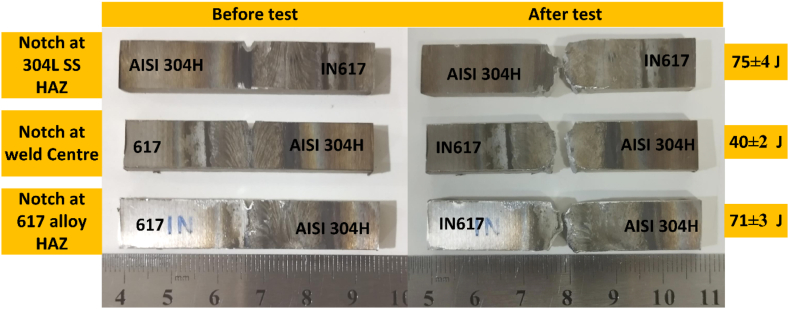
Impact test results of weldments.

**Fig. 16:**
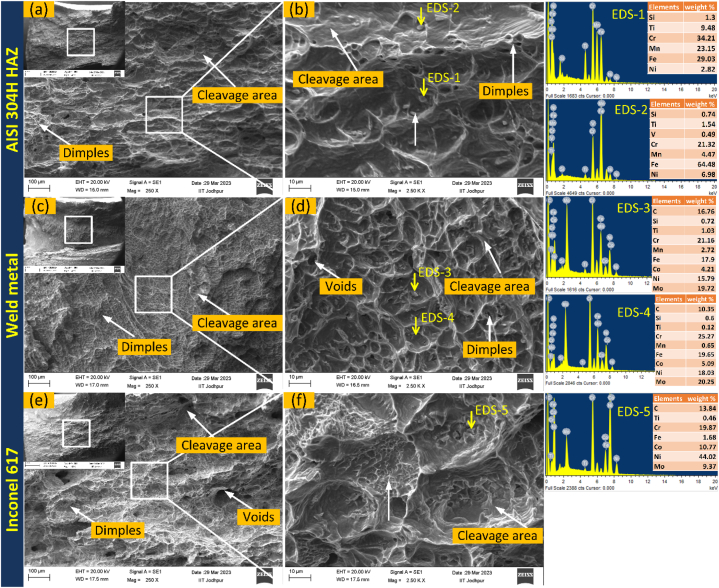
SEM image of fracture surface of impact tested specimen (a) AISI 304H HAZ, (b) weld metal, (c) IN617 HAZ.

The impact test results revealed that the TIG welded joint exhibited a weak impact toughness of 40 ± 2 J, which was lower than that of the AISI 304H HAZ (75 ± 4 J) and IN617 HAZ (71 ± 3 J). The test samples did not undergo any yielding during the impact loading and broke into two pieces, as shown in Fig. 15. The low impact strength of the weld metal may be attributed to the Mo-rich phases present along the inter-dendritic areas, which is consistent with previous research that has shown that segregation reduces the weld metal's impact resistance. YIlmaz and Tumer [44] reported analogous findings in their study of weldments involving AISI 316 L and AH36 steels. In terms of impact toughness, the AISI 304 HAZ emerged as the most resilient section of the dissimilar welded joint. The SEM fractography of the impact-tested samples validated the presence of microvoids, cleavage facets, and dimples, suggesting that the weld metal predominantly underwent a ductile-brittle mode fracture with cleavage dominance during impact loading (see Fig. 16(c, d)). These fracture surface features were in line with the impact test results (40 ± 2 J). The presence of the secondary phases (Mo_6_C and M_23_C_6_) was also inferred from the SEM/EDS (EDS 3/EDS 4). These phases could be a contributing factor to the weld metal's reduced impact energy absorption capacity. The failed AISI 304H HAZ impact-tested specimen exhibited larger dimples and significant cleavage areas on its fracture surface (Fig. 16(a, b)). EDS analysis (EDS 1/EDS 2) suggested the presence of Mn-rich particles, potentially MnS inclusions. Upon testing, the IN617 HAZ specimen also experienced fracture, breaking into two sections, and exhibited an impact toughness value comparable to that of the AISI 304H HAZ. An SEM examination of the fracture surface revealed Mo-rich secondary phases (EDS 5), dimples, microvoids, and cleavage areas (Fig. 16(e, f)).

## 4 Conclusions

1. A successful, well-bonded, defect-free joint was established for IN617 and AISI 304H. The detailed characterization of the interface demonstrated that the interface's sides were securely joined.
2. The inhomogeneous microstructure of the weld metal was observed in both optical and SEM images, which was a result of the localized cooling during solidification. The precipitates located within the inter-dendritic regions of the weld metal were identified through SEM/EDS analysis as being carbides of the M_23_C_6_ type, enriched with Cr and Mo, along with M_6_C carbides enriched with Mo. The Mo-rich phases found in the inter-dendritic spaces may also contribute to the low impact strength of the weld metal.
3. The HAZ of IN617 exhibited a notable increase in grain size and no PMZ was found at the interface. On the other hand, the HAZ of AISI 304H showed a minor change in grain size, but a significant PMZ and UZ were observed at the interface.
4. SEM/EDS confirmed that the major secondary phase in IN617 HAZ was Cr and Mo-rich M_23_C_6_ and Mo-rich M_6_C. Ni, Co, and Mo migration from IN617 BM to weld metal was confirmed by elemental line map and SEM/EDS. Within the AISI 304H HAZ, observations revealed the presence of polygonal equiaxed austenite grains, twin formations, and lath-shaped δ ferrite stringers. The migration of Fe from AISI 304H BM to weld metal was confirmed by the elemental line map.
5. According to the tensile test results, the failure was found to originate from weld metal near the interface of AISI 304H BM. Test samples that broke off from the weld metal showed a tensile strength value lower than that of the AISI 304H BM and IN617 BM.
6. The hardness plot showed a clear correlation between microstructure and hardness. The microhardness values gradually increased from AISI 304H to IN617 across the weld metal and interfaces. The hardness values at the interface of IN617 and AISI 304H measured 203 HV and 180 HV, respectively, while the weld metal exhibited a hardness of 199 ± 6 HV. The highest hardness value of 225 ± 4 HV was observed in IN617 HAZ.
7. The impact characteristics of the welded joint were negatively affected by the presence of Mo-rich phases in the weld fusion zone. In terms of impact strength, the IN617 HAZ exhibited the lowest performance among the weldments. The impact toughness was ranked in the following order: AISI 304H HAZ > IN617 HAZ > weld metal. The fracture surface SEM-EDS study of the weld metal confirmed the presence of Mo-rich secondary phases.

## Funding

No funding is received for the work.

## Author contribution statement

Sachin Sirohi: Contributed reagents, materials, analysis tools or data; Wrote the paper.

Amit Kumar: Analyzed and interpreted the data; Contributed reagents, materials, analysis tools or data; Wrote the paper.

S. M. Pandey; Dariusz Fydrych: Performed the experiments; Analyzed and interpreted the data; Contributed reagents, materials, analysis tools or data; Wrote the paper.

Priymbda Purohit: Analyzed and interpreted the data; Contributed reagents, materials, analysis tools or data.

Sanjeev Kumar: Conceived and designed the experiments; Performed the experiments; Analyzed and interpreted the data.

Chandan Pandey: Conceived and designed the experiments; Performed the experiments; Analyzed and interpreted the data; Contributed reagents, materials, analysis tools or data; Wrote the paper.

## Data availability statement

No data was used for the research described in the article.

## Declaration of competing interest

The authors declare that they have no known competing financial interests or personal relationships that could have appeared to influence the work reported in this paper.
